# Porcine ex-vivo intestinal mucus has age-dependent blocking activity against transmissible gastroenteritis virus

**DOI:** 10.1186/s13567-024-01374-y

**Published:** 2024-09-20

**Authors:** Waqar Saleem, Nathan Carpentier, Charlotte Hinnekens, Dayoung Oh, Sandra Van Vlierberghe, Kevin Braeckmans, Hans Nauwynck

**Affiliations:** 1 Laboratory of Virology, Department of Translational Physiology, Infectiology and Public Health, Faculty of Veterinary Medicine, Ghent University, Salisburylaan 133, 9820 Merelbeke, Belgium; 2 Polymer Chemistry & Biomaterials Group, Centre of Macromolecular Chemistry, Department of Organic and Macromolecular Chemistry, Ghent University, Krijgslaan 281, 9000 Ghent, Belgium; 3 Laboratory for General Biochemistry and Physical Pharmacy, Department of Pharmaceutics, Faculty of Pharmaceutical Sciences, Ghent University, Ottergemsesteenweg 460, 9000 Ghent, Belgium

**Keywords:** TGEV, intestinal mucus, single particle tracking, viral diffusion, age-dependent infection, infection block, mucus proteomics

## Abstract

Transmissible gastroenteritis virus (TGEV) causes high mortality in young piglets (< 3 days of age). With aging, the susceptibility/morbidity/mortality rates drop. We previously hypothesized that the age-related changes in the intestinal mucus could be responsible for this resistance. Hence, this study investigated the effect of porcine intestinal mucus from 3-day and 3-week-old pigs on the free mobility of the virulent TGEV Miller strain, and on the infection in swine testicle (ST) cells. Single particle tracking (SPT) revealed that TGEV had significantly higher diffusion coefficients in 3-day mucus compared to 3-week mucus. TGEV and charged and uncharged control nanoparticles diffused freely in 3-day mucus but were hindered by 3-week mucus in the diffusion model; TGEV mimicked the diffusion behavior of negatively charged carboxylated particles. Inoculation of ST cells with TGEV in the presence of 3-week mucus resulted in a significantly lower average number of infected cells (30.9 ± 11.9/5 fields) compared with 3-day mucus (84.6 ± 16.4/5 fields). These results show that 3-week mucus has a significant TGEV-blocking activity compared to 3-day mucus in free diffusion and infection of the underlying susceptible cells. Additionally, a label-free proteomics analysis revealed an increased expression of mucin 13, known for negatively regulating the tight junctions in intestinal epithelium, in 3-day-old pigs. In 3-week-old pigs, a higher expression of mucin 2, a type of secreted mucin which is known for inhibiting coronavirus infection, was observed. Concludingly, this study demonstrated a protective effect of 3-week mucus against viral infections.

**Supplementary Information:**

The online version contains supplementary material available at 10.1186/s13567-024-01374-y.

## Introduction

Viral gastroenteritis is a major challenge in pig industry, resulting in significant economic losses [1]. Porcine coronaviruses, mainly transmissible gastroenteritis virus (TGEV), porcine epidemic diarrhea virus (PEDV), porcine deltacoronavirus (PDCoV), and porcine rotaviruses cause high morbidity and mortality in pig farms all over the world [2]. These viruses infect the enterocytes of small intestines in pigs of all ages, causing diarrhea, vomiting, dehydration, and high mortality [3]. These clinical signs are very severe in young piglets, and the mortality rate can reach 70–100% in 1 to 3-day-old piglets without lactogenic immunity [4]. Commercial pig farms reduce the weaning time to 21–28 days to shorten the breeding period and maximize production, resulting in compromised lactogenic immunity and young pig’s gut health [5, 6]. The reasons behind severe infection and high mortality during the first few days of the piglet’s life are poorly understood.

TGEV is an enveloped, single-stranded positive-sense RNA virus of the genus *Alphacoronavirus* with a genome of ~ 28.5 kb [7], containing nine open reading frames (ORF), with ORF1a and ORF1b occupying 2/3rd of the whole genome, and are involved in its replication [8]. Among its 16 non-structural proteins (Nsps), Nsp1 mainly interferes with the host immune response [9], while Nsp7–16 form a variety of multi-protein complexes and are linked with severe disruptions of intestinal homeostasis [10, 11]. TGEV Spike (S) protein is a large transmembrane glycoprotein that helps to identify and bind host receptors, mainly aminopeptidase-N (APN) [12]. Still, recent studies have revealed TGEV Purdue replication in APN-negative cells [13]. The S protein undergoes multiple genetic alterations and recombination, resulting in the emergence of new strains, as evident from nt621–681 deletions, giving rise to Porcine Respiratory Coronavirus (PRCV) [14]. TGEV infects enterocytes of all ages, but the infection is more prominent in young piglets. Studies on its titer in porcine tissues of different ages suggest a higher infection rate in younger pigs (3-day-old) than in 3-week-old pigs after 3–4 days of infection [15, 16].

Intestinal mucus is the main barrier between nutrients, pathogens, and enterocytes. The small intestinal mucus layer is relatively thinner (25.9 ± 11.8 μm in the duodenum to 31.0 ± 15.7 μm the ileum) than in the colon (35.1 ± 16.0 μm in the descending colon). The colon has two layers: an epithelium adherent inner layer and a loose outer layer [17, 18]. Mucins (MUC), building blocks of mucus, are secreted from the Goblet cells. They have a protein backbone and *O*-linked oligosaccharide modifications [19]. Gel-forming mucins give viscoelastic properties to the mucus due to disulfide bonds at their cysteine-rich N- and C-terminals [20]. Among these, (i) MUC2 is the principal gel-forming mucin in the intestinal mucus barrier [21], (ii) MUC5AC is mainly produced in the stomach (an intestinal upregulation may be seen during enteric nematode infections) [22], (iii) MUC5B is expressed in the colon [23], (iv) MUC6 is present in the stomach and duodenum [23] and (v) MUC7 is found in saliva [24]. The mucus layer protects the intestinal mucosa against damage from host proteases and invading pathogens [25].

The mucus layer changes rapidly during the early stages of life [26]. These changes occur both in terms of mucus quantity and quality. Our previous study showed that the percentage of mucus-producing cells increased with age along the whole length of the porcine intestines from 3-day-old piglets to 3-month pigs [27]. A study on broiler chicks showed similar trends with an increasing number of intestinal goblet cells from embryonic day 19 to the 2-day-old chick [28]. By Single Particle Tracking (SPT) and Multiple Particle Tracking (MPT) techniques, trajectories of different nanoparticles and viruses within a medium of interest, such as ex-vivo intestinal mucus, can be analyzed to gain information about the interaction of these particles with this medium [29, 30]. A study on rats found that particle diffusion was faster in mucus from 5-day-old animals when compared to 21-day-old [31]. In another study, the diffusion of 500 and 1000 nm carboxylated particles through the mucus of 2-week-old pigs was higher than that of 7/10-month-old pigs. The authors suggested this may have occurred due to the increased DNA concentration in older animals’ mucus originating from shed epithelial cells [32]. This indicates that the age-related changes in intestinal mucus affect the behavior of particles and viruses, and mucus from older animals offers more hindrance than from newborns. We hypothesize now that porcine mucus plays a significant role in protecting older animals (> 3 weeks) against TGEV infection when compared to younger animals (3–5-day-old).

To test this hypothesis, (i) the mobility of TGEV particles was analyzed in ex-vivo intestinal mucus from 3-day and 3-week pigs using SPT, (ii) the diffusion of TGEV was examined in ex-vivo intestinal mucus from 3-day and 3-week pigs at different temperatures, (iii) the blocking effect of 3-day and 3-week ex-vivo intestinal mucus was tested on TGEV infection of ST cells, and (iv) to do proteomics on the mucus from the two age groups to identify differences in (muco)proteins. This work lays the groundwork to understand better the age-dependent changes in pigs’ intestinal mucus and the mucus barrier function against viral infections, which may finally lead to better combat enteric viral infections.

## Materials and methods

### Pigs

Pigs (Belgian Piétrain) were obtained from a high-health (no clinical signs), TGEV/PRCV-negative farm at three days and three weeks of age. The pigs were kept off-feed for 24 h and euthanized with pentobarbital (Kela, Hoogstraten, Belgium) at a dose of 12.5 mg/kg body weight. Experiments were repeated using three pigs from each age group (total *n* = 6).

### Ex-vivo intestinal mucus collection

Intestinal mucus (“ex-vivo mucus” from here on) was collected according to the protocol devised by Wheeler et al. [33]. Briefly, small intestines were collected from euthanized pigs from the duodenum to the ileocecal junction. The mesentery was gently removed, 20–30 cm pieces of intestines were cut and laid down longitudinally, and the luminal surface was exposed using a scalpel. Mucus was scraped gently using the back of the scalpel blade and solubilized (1 g scrapings in 5 mL) in 0.2 M sodium chloride buffer (pH = 7) with 5 mM benzamidine hydrochloride and 0.5 mM 4-benzenesulfonyl fluoride hydrochloride (both serine protease inhibitors), 1 mM dibromoacetophenone (cysteine protease inhibitor), 5 mM EDTA (metalloprotease inhibitor), and 0.04% sodium azide (Sigma). Cellular debris and food waste were removed using low-speed centrifugation at 4000 rpm for 30 min at 4 °C. The supernatant was aliquoted and stored at −20 °C.

### Fluorescent labeling of virus

TGEV was fluorescently labeled using the protocol devised by Yang et al. [34]. Swine testicle (ST) cells were inoculated with TGEV Miller (TCID_50_ 10^8.5^/mL) for 24 h. The medium was harvested, and the cellular debris was removed by centrifugation at 4000 rpm for 20 min at 4 °C. The virus was pelleted at 75 000 × *g* for 2 h at 4 °C in a type 45Ti rotor (Beckman Coulter Inc. CA, USA). The supernatant was discarded, and the pellet was resuspended in HNE buffer (5 mM HEPES, 150 mM NaCl, 0.1 mM EDTA, pH 7.4). DiO (3,3'-Dioctadecyloxacarbocyanine perchlorate; Vybrant™ DiO Cell-Labeling Solution; Invitrogen, Merelbeke, Belgium), a lipophilic dye that intercalates in the viral envelope and is visible in the 480/505 nm region [35], was used to label TGEV. Briefly, 2 nM of DiO was mixed with 50 µL of purified virus using a high-speed vortex. After 20 min of incubation at room temperature (RT), the mixture was passed through Microspin G-50 columns (Cytiva, USA), and the unlabeled dye was eluted. Labeled un-eluted virions were collected from columns, 4 µL was mounted on a glass slide, and the success of the virus labeling was confirmed by fluorescence microscopy (Leica DM RBE), using 1000× magnification. The titer of labeled virions was determined on ST cells. The effect of labeling on the size and surface charge (ζ-potential) of the labeled and unlabeled virions was analyzed using Zetasizer Nano ZS (Malvern Panalytical Ltd, UK) as previously described [36].

### Surface modification of nanoparticles

Nanoparticles were used as control particles in the present study. Surface modification of green-fluorescent carboxylated nanoparticles (100 nm, Thermo Fisher Scientific Inc. USA) was carried out using methoxy polyethylene glycol (mPEG) and 1,2-Dimethylethylenediamine (DMEDA) as described by Forier et al. [37]. Briefly, carboxyl groups on the surface of particles (2.7 × 10^13^ particles/mL) were activated by 4 mg/mL N-(3-dimethylaminopropyl)-N’-ethylcarbodiimide hydrochloride (EDC; Sigma-Aldrich) and 1.13 mg/mL N-hydroxysulfosuccinimide sodium salt (NHS, Sigma-Aldrich) in HBS buffer [10 mM HEPES (Sigma-Aldrich), 150 mM NaCl (Sigma-Aldrich)]. Subsequently, 20 mg/mL of mPEG (5 kDa) or 10 mg/mL of DMEDA was added to the mixture, and the pH was set at 8 or 5, respectively. The reaction mixtures were incubated overnight at 4 °C with a magnetic stirrer. The buffer and reagents were removed by filtering through Amicon® Ultra-4 Centrifugal Filter Units (100 kDa MWCO, Millipore, MA, USA) for 30 min at 3300 × *g*. After two washings with HBS buffer, the particles were resuspended in HBS (pH = 8) and stored at 4 °C. As discussed above, the size and ζ-potential of modified particles were also measured.

### “Short duration diffusion” analysis using SPT

SPT analysis was conducted as described by Yang et al. [38]. Briefly, the DiO-labeled TGEV suspension was diluted to 10^8^ TCID_50_/mL, and the modified control particles were diluted to 10^8^ particles/mL in ex-vivo mucus of both age groups and in Milli-Q® ultrapure water (Sigma) to be used as negative control. Press-to-seal™ silicone isolators (20 mm diameter, 0.5 mm deep, Invitrogen, Merelbeke, Belgium) were mounted on glass slides, and 4 µL of DiO-labelled TGEV or modified control particles in ex-vivo mucus or Milli-Q® ultrapure water were brought into the center of the isolators and covered gently by coverslips. The Brownian movement trajectories of fluorescent virions/particles in ex-vivo mucus were recorded by a fast and sensitive electron-multiplying charge-coupled device (EMCCD) camera (Cascade II: 512; Roper Scientific, Tucson, AZ, USA) mounted on an inverted epifluorescence spinning disk microscope (Nikon TE2000E, Nikon Belux, Brussels, Belgium), equipped with a 100 × oil-immersion objective (Plan Apochromat, Nikon). The movies were captured with the NIS Elements AR software (Nikon) at a temporal resolution of 46.2 ms for 5 s; hence short-duration diffusion. The illumination time was 15 ms per frame. Trajectories of *n* ≥ 500 particles were analyzed for each age-group, temperature, and time point, and at least three independent experiments were performed for each condition. Movies were analyzed with the Image Processing Software (IPS, in-house developed software) to extract XY positional data over time. The diffusion coefficient (*D*) was calculated as a function of each particle’s time scale (t).

### Mucus “long duration diffusion” experiment set-up

**Figure 1:**
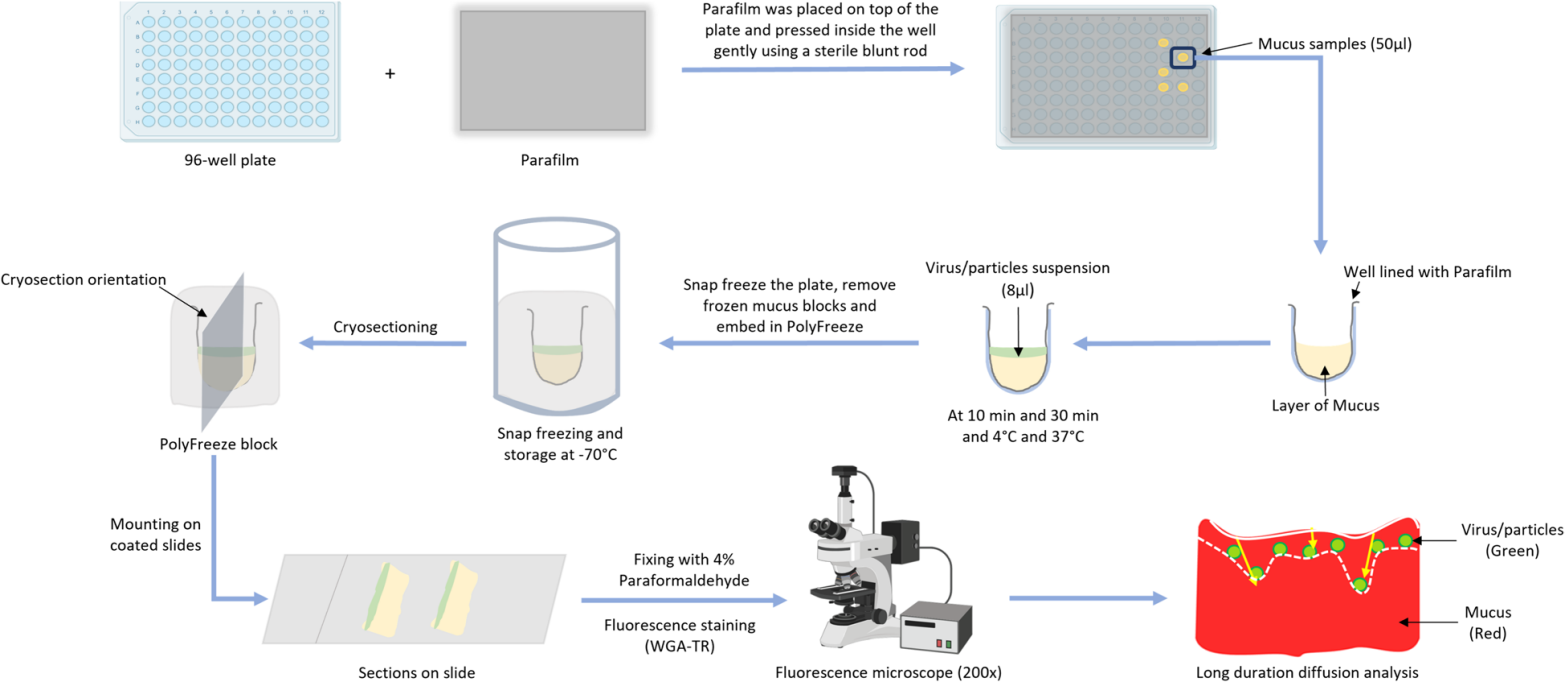
**Schematic representation of the experimental set-up for mucus “long-duration diffusion” analysis.**

An in-house experimental set-up was devised to study the diffusion of TGEV and particles through ex-vivo mucus for 10 or 30 min (hence termed long-duration diffusion), as shown in Figure 1. Briefly, a 96-well plate was covered on top by a piece of Parafilm®, which was gently pressed inside the wells using a sterile blunt rod so that the Parafilm® covered the inner linings of the well. Mucus (50 μL/well) from 3-day and 3-week pigs was filled in these lined wells of four separate plates; two plates were incubated at 4 °C and two at 37 °C. Subsequently, 8 μL of DiO-labelled TGEV (TCID_50_ 10^7.6^/mL), carboxylated, PEGylated, and amide-modified particles (each diluted to 10^8^ particles/mL) were gently put on top of the mucus layer of respective plates. One plate for each temperature was incubated for 10 min and the second for 30 min. The plates were snap-frozen in a dry ice/ethanol bath (−70 °C) for 5 min. The frozen mucus blocks (1–1.5 mm in height) were removed from the wells, immediately embedded in PolyFreeze Tissue Freezing Medium (Sigma), and snap-frozen in a dry ice/ethanol bath. All samples were stored at −70 °C till further use.

Six 10 µm longitudinal sections were made from all PolyFreeze blocks, using a CM 1950 cryostat at −20 °C (Leica Biosystems) with a trimming interval of ~ 100 µm between each section and loaded onto 3-aminopropyltriethoxysilane-coated (Sigma-Aldrich, St. Louis, MO, USA) glass slides. The cryosections were fixed in 4% paraformaldehyde for 10 min at RT. To stain mucus, the sections were incubated with 10 µg/mL Wheat-germ Agglutinin-Texas Red conjugate® (WGA-TR; ThermoFischer) for 20 min at RT, washed three times with PBS and mounted with glycerol-DABCO. WGA is a lectin that has an affinity to bind to the sialic acid residues present in mucins [39]. Images were taken using a Leica DM RBE fluorescence microscope at 200× magnification. ImageJ (NIH, USA) was used to measure the distance of virus/particle diffusion at ten different regions per section from the solid line to the dotted line, as shown in Figure 1, and the average was taken. This was repeated for all the sections per animal, temperature, and time point and collective averages were taken. The experiments were repeated three times with mucus from different animals per age group for statistical analysis.

### Immuno-peroxidase monolayer assay (IPMA)

To test the blocking effect of ex-vivo mucus on TGEV replication in ST cells, an IPMA was designed by modifying the protocol by Haegeman et al. [40]. ST cells were seeded in 96-well plates at 200 000 cells/mL and allowed to grow and differentiate for 72–96 h. Mucus “overlay” and mucus “inoculum” setups were designed to see the post-infection and pre-infection blocking effects, respectively. In the “overlay” setup, TGEV Miller strain (10^8.5^ TCID_50_/mL stock concentration) was diluted 100 × in Dulbecco’s Modified Eagle Medium (DMEM), supplemented with 10% Fetal Bovine Serum (FBS); 100 U/mL penicillin (Gibco, Grand Island, NY, USA), 0.1 mg/mL gentamicin (Gibco, Paisley, UK), 0.1 mg/mL streptomycin (Gibco, Grand Island, NY, USA), and 5 μg/mL amphotericin B (Gibco, Grand Island, NY, USA), to finally get 10^6.5^ TCID_50_/mL. For overlays, 50% or 5% mucus overlays were prepared by mixing ex-vivo mucus from 3-day or 3-week pigs with 2 × Minimal Essential Medium (MEM) supplemented with 20% FBS, 200 U/mL penicillin (Gibco, Grand Island, NY, USA), 0.2 mg/mL gentamicin (Gibco, Paisley, UK), 0.2 mg/mL streptomycin (Gibco, Grand Island, NY, USA), and 10 μg/mL amphotericin B (Gibco, Grand Island, NY, USA). For positive control, a carboxymethylcellulose (CMC) overlay was prepared by mixing 1.88% CMC stock solution with 2 × MEM media in a 1:1 ratio to get a 0.94% final CMC concentration. For negative control, DMEM was used as an overlay. ST cells were inoculated with the virus for 1 h at 37 °C with 5% CO_2_, followed by removal of the inoculum and three washings with phosphate buffer saline with Ca^2+^ and Mg^2+^. This was followed by gently putting 100 µL of mucus/CMC/DMEM overlays on the cells. For the “inoculum” setup, TGEV (10^8.5^ TCID_50_/mL) was diluted 100 × with 50% and 5% mucus, 0.94% CMC, or DMEM, as mentioned above, to get final concentrations of 10^6.5^ TCID_50_/mL for each inoculum. The inoculums were incubated at 37 °C for 30 min before 100 µL of each was added to thrice-washed TGEV-infected ST cells. The “overlay” and “inoculum” plates were incubated for 24 h at 37 °C with 5% CO_2_.

After incubation, the ‘overlay’ and ‘inoculum’ mixtures were removed, and cells were washed twice with PBS (100 μL/well) and dried for 1 h at 37 °C. Then, the plates were frozen at −20 °C for at least 2 h or overnight. The cells were then fixed with 4% paraformaldehyde (100 μL/well) for 10 min, followed by two washings of PBS for 5 min each. Intrinsic peroxidase activity was inhibited by adding 100 μL freshly made 30:1 methanol/33% H_2_O_2_ mixture. After 5 min of incubation at RT, the cells were rewashed twice with PBS. Cells were then incubated with TGEV/PRCV hyperimmune serum (1:1000, raised in-house against PRCV 91V44 strain), diluted in salt-PBS, containing 0.1% Tween 80 and 10% negative goat serum (50 μL/well) for 1 h at 37 °C. After two washings with PBS containing 0.1% Tween 20 (100 μL/well), cells were incubated with goat anti-swine IgG peroxidase (1:500), diluted in salt-PBS, comprising 0.1% Tween 20 and 10% negative goat serum (50 μL/well) for 1 h at 37 °C. After washing twice again with PBS containing 0.1% Tween 20 (100 μL/well), 50 μL/well of a substrate solution [3-amino-9-diethyl-carbazole (AEC) in 50mM Na-acetate buffer (pH = 5) with 0.05% H_2_O_2_] was added to reveal the infected cells. The mixture was incubated at room temperature for 15–20 min. The reaction was stopped by removing the substrate and adding 100 μL/well of the Na-acetate buffer. Finally, the number of positive cells in five random fields per well was counted using an Olympus IX50 inverted phase-contrast microscope (Olympus Life Science, Tokyo, Japan) at 200×, and the average number per five fields was calculated. This was repeated three times for each age-group and experimental condition using different animals.

### Proteomic analysis

Label-free proteomics was performed on the ex-vivo intestinal mucus samples from 3-day pigs (*n* = 3) and 3-week pigs (*n* = 3) in the VIB Proteomics core facility (Vlaams Instituut voor Biotechnologie, Ghent, Belgium). Liquid Chromatography with tandem mass spectrometry (LC–MS/MS) runs of all samples were searched collectively, using the DiaNN algorithm (version 1.8.1) with mainly default search settings, including a false discovery rate set at 1% on precursor and protein level. Spectra were searched against the Sus scrofa protein sequences in the Uniprot database (database release version of 2024_01) [41], containing 22 786 sequences and the common contaminants protein sequences database [42]. Missing values due to low expression of analyte species, a common occurrence in omics analysis, were imputed from the sample’s “noise level” feature intensity distribution. However, for specific protein analysis, only the proteins with complete brackets in all three replicates per age group were selected. Hierarchical clustering in proteomics allows the grouping of proteins based upon their expression pattern [43]. Using algorithms like Euclidean distance, similarity between the expression pattern of proteins of different samples can be estimated by using straight-line distances between two points in the form of dendrograms [44]. Heat maps were created by transforming log_2_ MaxLFQ values of identified proteins into z-scores, using Morpheus to perform hierarchical clustering under Euclidean distance metric, average linkage method, and clustering both rows and columns [45].

### Statistical analysis

The two-way analysis of variance (ANOVA) followed by Tukey’s and Šídák’s multiple pairwise comparison post hoc tests were performed using GraphPad Prism 9.0 (GraphPad Software, Inc., San Diego, CA, USA). Individual mean values with standard deviation (SD) of three independent experiments were represented as scatter graphs with bars, and results with *p* < 0.05 were considered significant. For SPT, the distribution coefficients (*D*) were further refined with a maximum entropy method (MEM) [46] to improve the precision of the distributions and remove features that are not statistically acceptable. For proteins identified through label-free proteomics, statistical differences between the two age groups were determined by Limma [47], and the statistical significance for differential regulation was set at False Discovery Rate (FDR) < 0.05 and |log_2_FC|≥ 2.

## Results

### DiO-labeled TGEV and modified particle characterization

**Figure 2:**
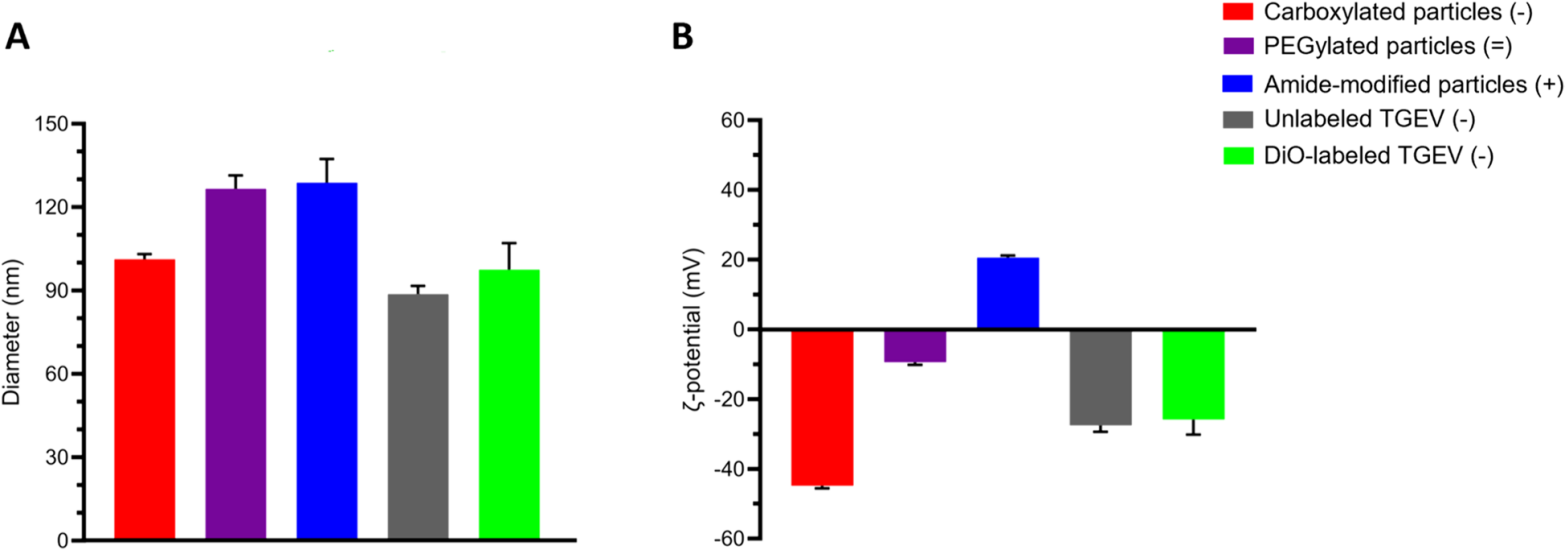
**Characterization of modified particles and labeled and unlabeled virions**. **A** Mean diameter values ± SD of three independent readings of modified particles, labeled TGEV and unlabeled TGEV, are plotted. **B**: Mean ζ-potential values ± SD of three independent readings of modified particles, labeled TGEV and unlabeled TGEV, are plotted.

Particle/virus size (nm) and surface charge in terms of ζ-potential (mV) were measured using Zetasizer Nano ZS (Malvern Panalytical Ltd, UK). Figure 2 indicates the properties of particles and labeled and unlabeled virions. PEGylated and amide-modified particles increased in size compared to the carboxylated particles (Figure 2A). PEGylated particles exhibited a decreased negative surface charge compared to carboxylated particles and were considered “near neutral” for subsequent experiments. Amide-modified particles showed a positive surface charge (Figure 2B). These particles will be represented as “carboxylated (−)”, “PEGylated (=)”, and “amide-modified (+)” while the virus as “TGEV (−)” from here on.

### SPT analysis showed that TGEV moved more freely in 3-day mucus as compared to 3-week mucus at both temperatures

**Figure 3:**
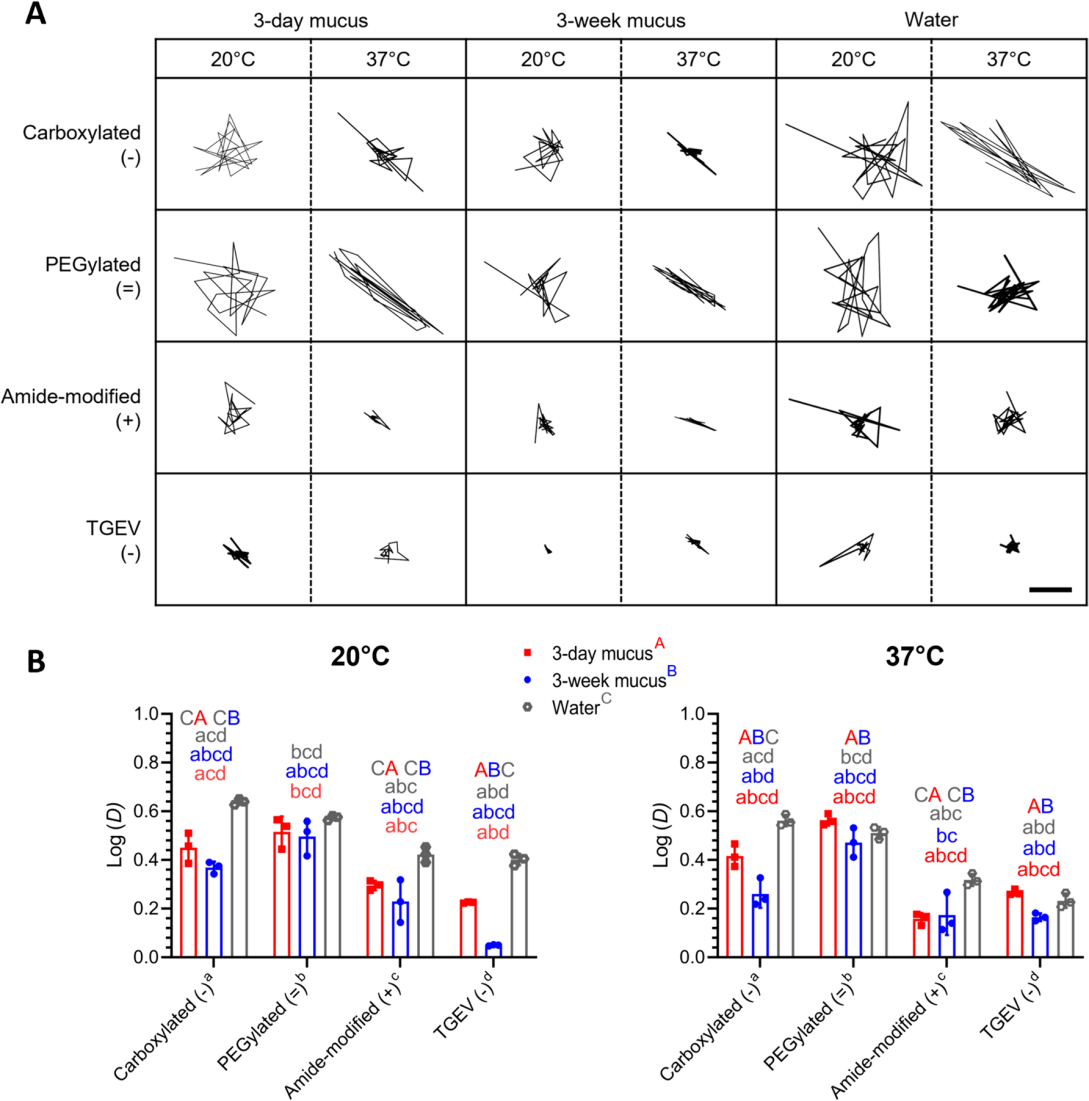
**SPT analysis of modified particles and labeled TGEV in 3-day and 3-week mucus at 20 °C and 37 °C. A** Representative trajectories of particles/virions in 3-day and 3-week mucus at 20 °C and 37 °C recorded for 5 s (Scale bar = 2 µm). **B** Individual mean logarithmic values of diffusion coefficient (*D*) with MEM ± SD of three animals per age group are plotted as scatter graphs with bars. Statistically significant differences were determined by two-way ANOVA. Small alphabets (a, b, c, d) represent significant differences (*p* < 0.05) between particles/TGEV (Tukey’s multiple comparison post hoc test). Capital alphabet groups (A, B, C) represent significant differences (*p* < 0.05) between 3-day mucus, 3-week mucus, or water (Šídák’s multiple comparison post hoc test). For detailed comparisons between modified particles and labeled TGEV in 3-day and 3-week mucus at 20 °C and 37 °C, refer to Additional file 1 (Figure 3; Annex A and B).

Modified particles and labeled TGEV were tracked using SPT in 3-day and 3-week mucus at 20 °C and 37 °C, and water was used as a negative control for a short duration of 5 s. Figure 3A shows the representative trajectories of modified particles and TGEV in 3-day mucus, 3-week mucus, and water recorded over 5 s at 20 °C and 37 °C. The diffusion coefficient (*D*) was calculated using IPS and refined using MEM analysis, and logarithmic values of *D* were used in subsequent statistical analysis (Figure 3B). Carboxylated (−), PEGylated (=), and TGEV (−) at 20 °C, while only TGEV (−) at 37 °C, had significantly higher log (*D*) values in 3-day mucus compared to 3-week mucus (Figure 3B; *p* < 0.05). At 20°C, TGEV (−) had significantly lower log (*D*) values than carboxylated (−) and PEGylated (=) particles in 3-day mucus and 3-week mucus, while significantly lower than amide-modified (+) particles in only 3-week mucus (Figure 3B-left; *p* < 0.05). At 37 °C, the TGEV (−) log (*D*) value was significantly higher than amide-modified (+) particles in 3-day mucus (Figure 3B; 0.27 ± 0.012 versus 0.16 ± 0.024, *p* = 0.017). Similar to what was observed at 20 °C, TGEV (−) had significantly lower log (*D*) values than carboxylated (−) and PEGylated (=) particles in 3-day mucus and 3-week mucus (Figure 3B-right; *p* < 0.05). In mucus from both age groups and water, the behavior of PEGylated (=) particles was similar and statistically non-significant (Figure 3B; *p* > 0.05). From these observations, it can be concluded that both carboxylated (−) and PEGylated (=) particles showed a higher diffusion than amide-modified (+) particles and TGEV (−). It can also be established that TGEV diffused more freely in 3-day mucus compared to 3-week mucus at both temperatures.

### Particles and TGEV diffuse freely in 3-day mucus at both 4 °C and 37 °C

**Figure 4:**
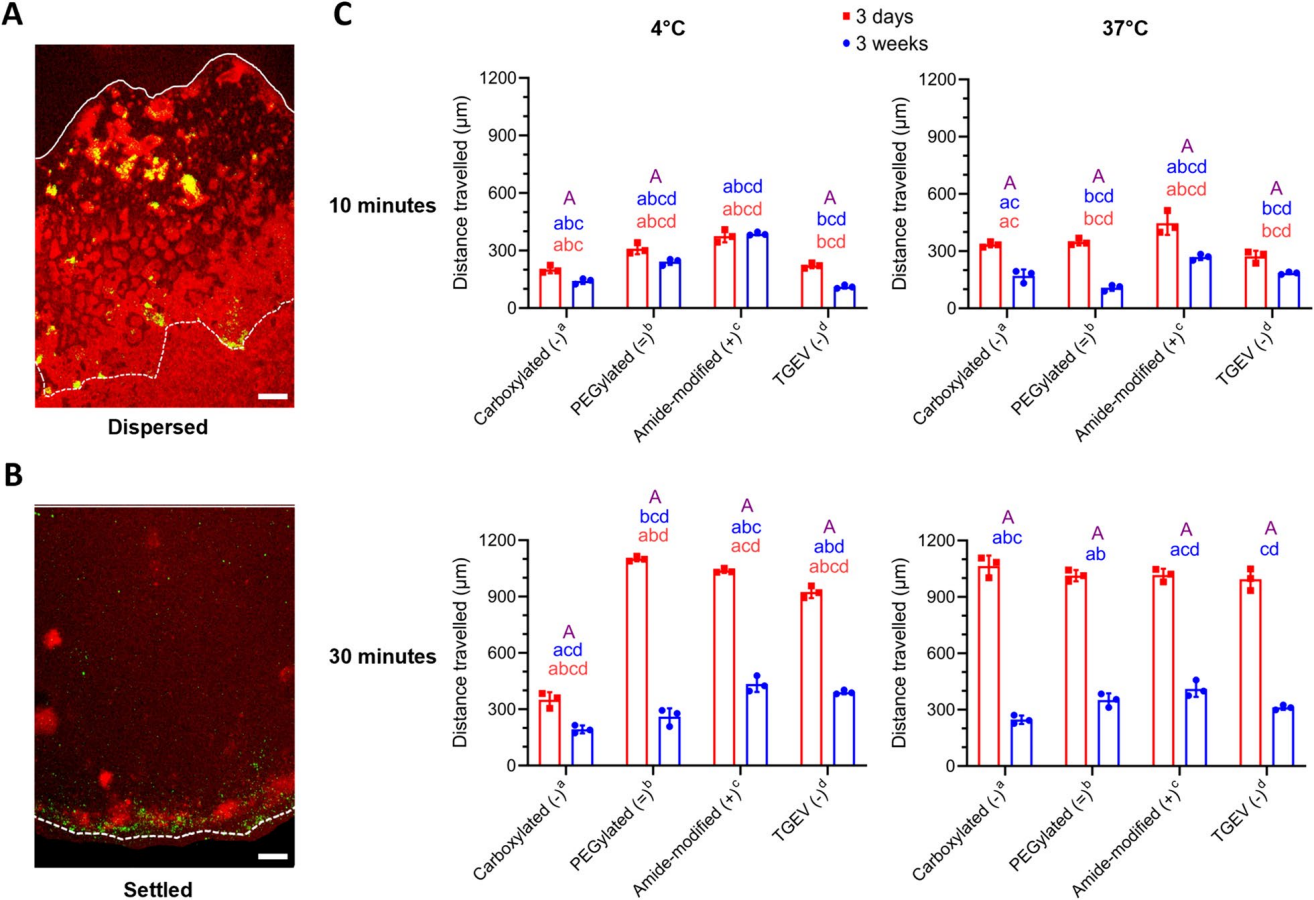
**Diffusion of modified particles and labeled TGEV in 3-day and 3-week mucus at 4 °C and 37 °C. A** Representative picture of diffused particles (green) in mucus (red) in the “dispersed” stage (scale bar = 50 µm). **B** Representative picture of diffused particles (green) in mucus (red) in the ‘settled’ stage (scale bar = 50 µm). **C:** Individual mean diffusion values ± SD of three animals per age group at 4 °C and 37 °C at 10 and 30 min are plotted as scatter graphs with bars. Statistically significant differences were determined by two-way ANOVA. Small alphabets (a, b, c, d) represent significant differences (*p* < 0.05) between particles/TGEV (Tukey’s multiple comparison post hoc test). Capital “A” means significant differences (*p* < 0.05) between age groups (Šídák’s multiple comparison post hoc test). Detailed diffusion comparisons between the age groups and particles/virions at 10 and 30 min are presented in Additional file 1 (Figure 4; Annex A and B).

Modified particles and labeled TGEV were allowed to diffuse through 3-day and 3-week mucus at 4 °C and 37 °C, and measurements were taken after 10 and 30 min (long-duration diffusion analysis). Figure 4A shows representative pictures of diffused particles in dispersed form. After 30 min, all particles and virions [except carboxylated (−) at 4 °C] settled already at the bottom of the mucus block (Figure 4B; average height of mucus block was ~ 1200 µm). In general, the particles moved faster than TGEV in mucus. All modified particles and TGEV diffused significantly better in 3-day mucus than in 3-week mucus at both temperatures (Figure 4C; *p* < 0.05), except amide-modified (+) at 4 °C (Figure 4C; 375.9 ± 32.38 µm versus 387.8 ± 8.77 µm, *p* = 0.92). After 10 min, TGEV (−) and carboxylated (−) particle diffusion was significantly lower than amide-modified (+) and PEGylated (=) particles in both 3-day and 3-week mucus at both temperatures (Figure 4C; *p* > 0.05). After 30 min at 4 °C, TGEV diffusion was significantly more compared to carboxylated (−) and PEGylated (=) in both 3-day and 3-week mucus (Figure 4C; *p* < 0.05). However, after 30 min at 37 °C, the TGEV exhibited decreased diffusion, which non-significantly differed with carboxylated (−) and PEGylated (=) particles (Figure 4C; *p* > 0.05). It can be concluded that 3-day mucus offers less diffusion hindrance for particles and TGEV at 4 °C and 37 °C than 3-week mucus.

### 3-week mucus significantly blocked the TGEV infection in ST cells, mimicking CMC

**Figure 5:**
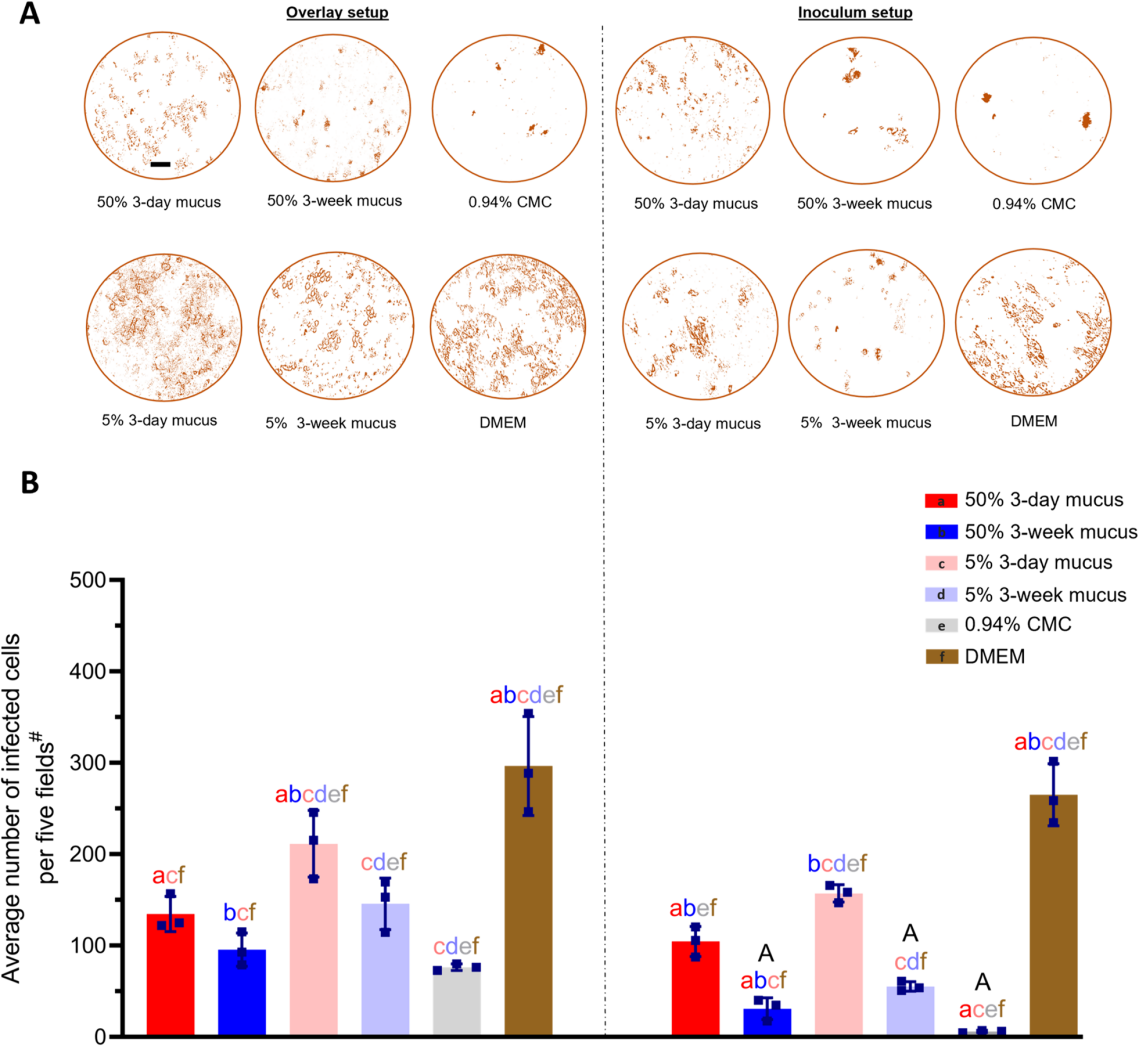
**“Overlay” and “inoculum” setups of mucus, CMC, and DMEM on TGEV-infected ST cells. A** Representative pictures of TGEV-infected ST cells in overlay and inoculum setups (scale bar = 50 µm). **B** Individual average means of infected cells ± SD of three animals per age group or control condition are plotted as scatter graphs with bars. Statistically significant differences were determined by two-way ANOVA. Small alphabets (a, b, c, d, e, f) represent significant differences (*p* < 0.05) between mucus samples, CMC, or DMEM within “overlay” or “inoculum” setups (Tukey’s multiple comparison post hoc test). Capital “A” represents significant differences (*p* < 0.05) between mucus samples, CMC, or DMEM in the ‘inoculum’ setup compared to their counterparts in the “overlay” setup (Šídák’s multiple comparison post hoc test). Detailed comparisons within and between overlay and inoculum setups of mucus, CMC, and DMEM on TGEV-infected ST cells are presented in Additional file 1 (Figure 5; Annex A and B). ^#^ refers to the five fields per well in which quantitation was performed.

A modified IPMA was designed to see the TGEV infection-blocking effect of 3-day and 3-week mucus on ST cells with CMC and DMEM used as positive and negative blocking controls, respectively. Figure 5A shows representative pictures of TGEV-infected ST cells in both “overlay” and “inoculum” setups 24 h after inoculation. In the “overlay” setup, there was a nonsignificant difference between the average number of infected cells per five fields for the 50% 3-day mucus (134.6 ± 15.72 cells) and 50% 3-week mucus (95.67 ± 18.1 cells) (Figure 5B-overlay setup; *p* = 0.42). However, in the “inoculum” setup, this difference was significant, with an average of 104.6 ± 13.36 infected cells per five fields for the 50% 3-day mucus and 30.90 ± 9.71 cells for the 50% 3-week mucus (Figure 5B-inoculum setup, *p* = 0.015). Comparisons were also made with a lower concentration of mucus (5%) in overlay and inoculum setups, but only 5% 3-day mucus in overlay setup had a significant difference with 50% 3-day mucus (Figure 5B,* p* < 0.05). Compared with the 0.94% CMC, 50% 3-week mucus had a non-significant difference in overlay and inoculum setups (Figure 5B,* p* > 0.05). When the two setups were compared, the average number of infected cells was significantly lower in the “inoculum” setup in both 50% 3-week mucus (Figure 5B; *p* = 0.024) and 5% 3-week mucus (Figure 5B; *p* = 0.01) as compared to the overlay setup. These results indicate that the 3-week mucus significantly blocks the TGEV infectivity in ST cells compared to the 3-day mucus, mimicking the effect of CMC, a commonly used plaque-blocking agent in virology [48]. Furthermore, infection blocking is more prominent in the “inoculum” setup, where the virus was first incubated with the mucus or CMC before infecting the cells.

### MUC13 was upregulated in 3-days mucus while MUC2 was upregulated in 3-week mucus

**Figure 6:**
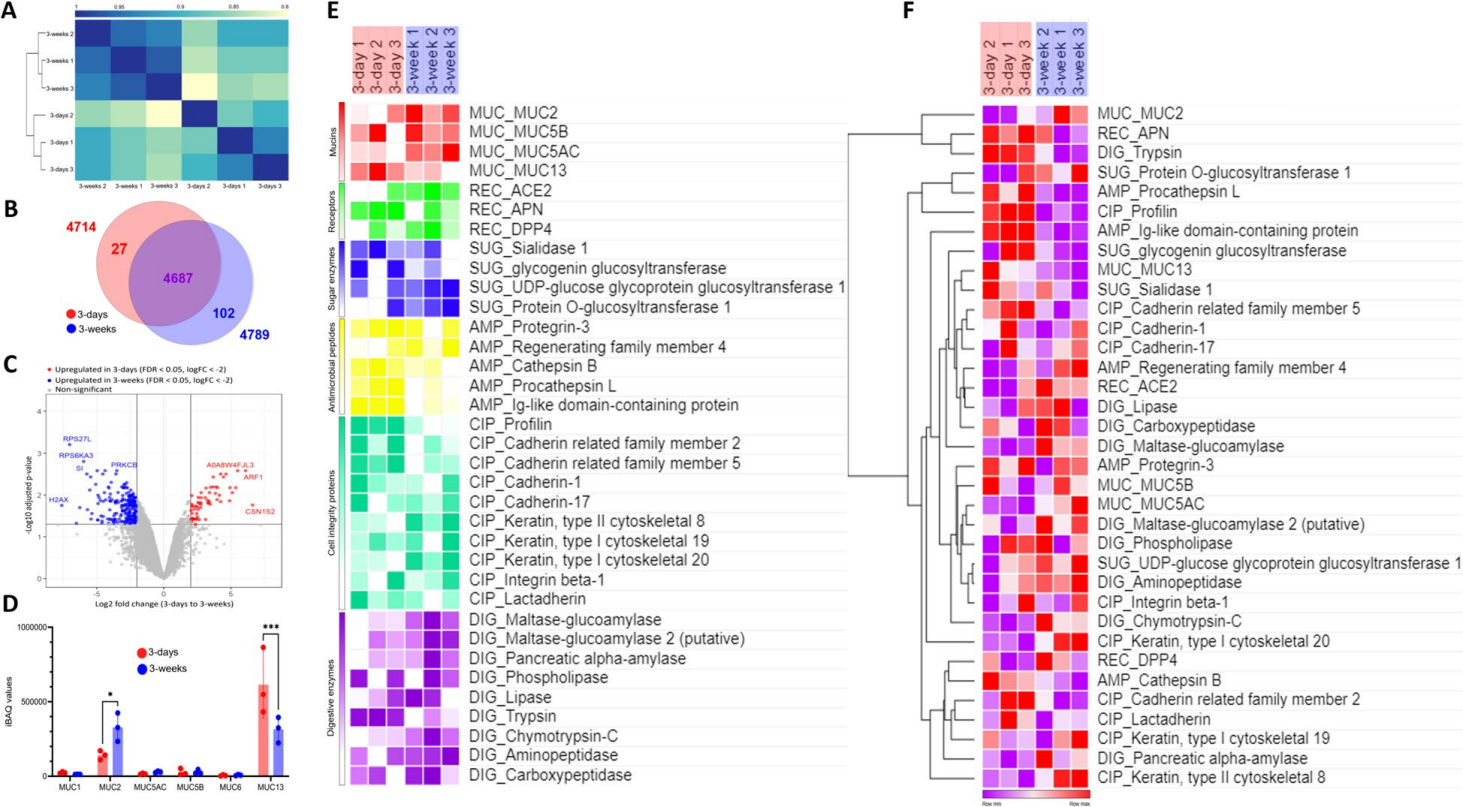
**Label-free proteomics analysis of intestinal mucus from 3 animals per age group**. **A** Heatmap of sample correlations after hierarchical clustering using Spearman correlation coefficients between protein expression values of all pairs of samples. **B** Venn diagram showing the unique and shared proteins between 3-day old pig and 3-week-old pig determined by the label-free analysis. **C** A volcano plot was generated for every protein identified using a log_2_ fold change of 3-days/3weeks on the X-axis, and -log_10_ adjusted p-value on the Y-axis. **D** Mean iBAQ values ± SD of identified mucins from the mucus of three animals per age group are plotted as scatter graphs with bars. Statistically significant differences, determined by two-way ANOVA (followed by Šídák’s multiple comparison post hoc test), are represented as *(*p* < 0.05) or ***(*p* < 0.001). **E** Heatmap showing the differences of z-scored values of the abundance of selected proteins from six categories (mucins, coronavirus receptors, sugar transferases, antimicrobial peptides, cell integrity proteins, and digestive enzymes) between each sample across 6 replicates (3 from each age-group). **F** The hierarchical clustering of selected proteins from six categories and 6 replicates across two age-groups using Euclidian distance is shown in the heat map. For both **E** and **F**, the heat map color variations shows the maximum and minimum protein abundance between each replicate of the two age groups (row min/row max).

Label-free LC–MS/MS runs of all samples identified 82 395 precursor peptides; 5679 protein groups were reliably quantified by intensity-based absolute quantification (iBAQ). Detailed iBAQ values can be found in Additional file 2 (sheet 1). Between-sample Spearman correlation showed similarities between 3 replicates of 3-days mucus and 3 replicates of 3-week mucus (Figure 6A). Figure 6B shows the shared and unique proteins in both age groups in the form of a Venn diagram. Out of 5679 total identified proteins, only those proteins which were expressed on all replicates per age group were taken. In Figure 6C, pairwise comparison between the means of the two groups is expressed as a volcano plot. In 3-days mucus, Ig-like domain proteins (A0A8W4FJL3), ADP-ribosylation factor 1 (ARF-1), Alpha-S2-casein (CSN1S2), and 56 other proteins were significantly upregulated. In 3-week mucus, Histone (H2AX), Ribosomal protein (RPS27L), Protein kinase C (PRKCB), and 230 other proteins were significantly upregulated. Details about these proteins can be found in Additional file 2 (sheet 2). Figure 6D shows all the mucins identified, with the highest expression of MUC13 followed by MUC2; MUC13 expression was significantly higher in 3-day mucus (*p* = 0.0003) while MUC2 was significantly higher in 3-week mucus (*p* = 0.03). In Figure 6E, the heatmap shows the relative expressions of selected proteins from different clusters between the six replicates across two age groups. Coronavirus receptors ACE2 and DPP4 were more expressed in 3-week mucus, while APN was more expressed in 3-day mucus. Antimicrobial peptides and cadherins were more expressed in 3-day mucus, while cytoskeletal keratin was more expressed in 3-week mucus. Digestive enzymes like maltase, amylase, lipase, amino- and carboxypeptidase were also more expressed in 3-week mucus. In Figure 6F, all these selected proteins were clustered using Euclidean distance, where expression pattern of mucins was dispersed, while those of cell integrity proteins and digestive enzymes was similar.

## Discussion

The pig industry suffers from viral gastroenteritis yearly [1]. TGEV is one of the main culprits in this regard. Although its detection rate has declined recently, and a new, less lethal PRCV has gained an endemic status [7], TGEV is still a significant problem in many parts of the world [49]. The mortality rate can reach 100% in young piglets (< 3 days of age) infected with TGEV [3], and the mechanism behind this age-dependent susceptibility is poorly understood. In our previous study, we concluded that this age-dependent susceptibility is not dependent on the expression level of known coronavirus receptors (APN, DPP4, ACE2, and TMPRSS2); instead, the number of mucus-producing cells in the intestines increased with age, suggesting a crucial role of intestinal mucus in peri-weaning and growing pigs against enteric infections [27]. Hence, this study aimed to show the TGEV-blocking effect of mucus from older peri-weaning pigs (3 weeks) compared to younger pigs (3 days), using in vitro approaches.

The surface electric charges and size of particles are of great importance in terms of their freedom to migrate through mucus due to the chemical bindings between the particles and mucus and the hindrance of the 3D network of mucins [50, 51]. Various drug delivery studies have modified the surface properties of commercially available nanoparticles, acknowledging particle size's limited role and particle surface charge's substantial role in penetrating the mucus barrier [52, 53]. It is not easy to mimic the surface characteristics of a virus within a synthetic nanoparticle, but bringing the overall charge to a near neutral can improve mucus permeation in vitro [54]. Small non-enveloped viruses like Norwalk virus (38 nm) and human papillomavirus (55 nm) penetrate mucus unrestrictedly as compared to the larger enveloped viruses because of their small size, absence of an envelope, and presence of capsid proteins, giving them an overall neutral surface [55]. TGEV, on the other hand, has a slightly larger size (80–120 nm) and a highly negative surface charge due to its host-derived lipid bilayer envelope carrying S, M, and E proteins [56], as evidenced in this study. As limited manipulation can be performed with a viral particle without losing its properties [57], stable negatively, positively, and neutral-charged polystyrene nanoparticles (~ 100 nm diameter) were included as controls in this study. PEGylation is used to modify commercial nanoparticles to decrease particle adhesion to mucin fibers and bring the overall charge to near neutral [53, 58]. In this study, we also utilized mPEG (5 kDa) to neutralize the surface of commercially available carboxylated nanoparticles, and the surface charge increased from -44.8 mV to -9.4 mV. Furthermore, we used DMEDA to modify carboxylated nanoparticles' amide to increase the charge to 20.6 mV.

The “short-duration diffusion” of modified particles and labeled TGEV was examined with a single-particle tracking analysis in the mucus of 3-day and 3-week-old pigs, and water was used as a control. In this technique, particle motion is recorded continuously for a short period (5 s) to generate a trajectory, and by statistically analyzing the multiple courses, it can reveal the diffusion pattern, adsorption–desorption and folding–unfolding dynamics, and trapping of particles in the biological fluids [59]. SPT revealed that the TGEV (−) was significantly blocked in 3-week mucus compared to 3-day mucus at 20 °C and 37 °C. Macierzanka et al*.* also saw a similar pattern of 500 nm and 1 µm carboxylated beads, blocked in intestinal mucus from older piglets but diffused freely in mucus from newborn piglets [32]. It was expected that the carboxylated (−) particles and TGEV (−) would behave the same in water. However, SPT analysis showed that the carboxylated (−) particles diffused more than TGEV (−). As the Brownian movement is primarily dependent on the size of particles and temperature of the medium [60], both of which were similar in our study, we suggest that the hydrophobic nature of polystyrene surface of carboxylated particles [61] and the hydrophilic nature of the virus lipid bilayer envelope [62] played a role in this variation. Furthermore, the Brownian movement of particles dispersed in fluids should increase with increasing temperature [63]. Still, we saw a decrease in the log (*D*) values of modified particles and labeled TGEV at 37 °C in mucus and water compared to 20 °C. Lock et al. described that the mucus structure varies with pH [64]. An increase in temperature decreases the biological pH due to the release of H^+^ ions, causing gelation [65] and possibly offering more hindrance to the particles to move at a higher temperature. These variations emphasize the impact of the physiochemical properties of mucus while carrying out these studies.

A “long-duration diffusion assay” was also developed to gain the mechanistic understanding of free diffusion of TGEV through porcine intestinal mucus from 3-day and 3-week-old pigs at different temperatures and time points. We observed that all modified particles and TGEV were hindered significantly in 3-week mucus compared to 3-day mucus at 4 °C and 37 °C. A similar study using somewhat bigger carboxylated-polystyrene nanoparticles (500 nm diameter) revealed that 99.4% of particles were blocked in ex-vivo intestinal mucus collected from adult pigs compared to around ~ 70% in mucus collected from infant pigs [32]. In the current study, PEGylated (=) and amide-modified (+) particles diffused the best in 3-day mucus at both temperatures. In another study, neutral polyelectrolyte particles (334 nm) and positively charged polyelectrolyte particles (144 nm) also diffused better than negatively charged polyelectrolyte particles (204 nm) in porcine intestinal mucus with diffusion coefficients of 0.029, 0.032 and 0.018 cm^2^/s respectively [50]. Furthermore, in our study, the TGEV (−) diffusion pattern was like the carboxylated (−) particles in 3-day and 3-week mucus at 37 °C but not at 4 °C. This is logical based on the similar net surface charges of both particles. Interestingly, SPT analysis revealed that the log (*D*) value of TGEV in 3-week mucus at 37 °C behaved more like amide-modified (+) particles. This difference can be attributed to the differences between the two analysis technologies. Indeed, with the SPT analysis, short time (5 s) and chaotic movements are measured, whereas with the “long duration diffusion”, long periods (10 and 30 min) and long-distance movements are analyzed. The chaotic Brownian movement seems very much related to the general changes on the surface of the particles. The “long duration diffusion” does not seem influenced by the general charges. Due to the limitation of the stage-top incubator for the spinning disk microscope, SPT analysis could not be performed at 4 °C.

To check the age-dependent blocking effect of ex-vivo intestinal mucus on TGEV infection in susceptible ST cells, an IPMA was modified with “overlay” and “inoculum” setups to compare the post-infection and pre-infection blocking effects. Compared to the negative control DMEM, the blocking results for mucus from both age groups were significant, showing the infection-blocking activity of mucus in general. However, when TGEV was incubated with the 3-week mucus (50% and 5% concentrations) before inoculation, the number of infected ST cells was significantly less than that of 3-day mucus. Iseli et al*.* also developed a similar assay to check the influenza A virus-blocking effect of human respiratory mucus by incubating the virus with different concentrations of mucus before inoculation. They compared the results with virus-MEM-infected cells. The results were significant in a dose-dependent manner [66]. Even diluting the 3-week 10× did not significantly decrease the blocking activity in overlay and inoculum setups. Compared to the positive control (CMC), 3-week mucus had a non-significant difference in both setups, showing high blocking activity compared to the 3-day mucus. Thus, it can be concluded that the physiochemical properties of pigs’ intestinal mucus considerably change during the first few weeks of life and offer much room for investigating age-dependent viral infections. Furthermore, this method of checking virus infection-blocking can also be adapted for various mucus sources and viruses.

The method used for mucus collection in this study is convenient, and intrinsic proteolytic activity is largely diminished due to the addition of a cocktail of protease inhibitors [33]. However, this causes an unavoidable dilution factor to the mucus, which can influence the results. Macierzanka et al*.* described and analyzed another method in which fresh mucus was scraped from the intestines of pig intestines within 20 min of slaughtering. Experiments were carried out within 30 min of mucus collection [67]. The current study adopted this method, but the cellular and intestinal debris offered problems in the subsequent experiments, and the urgency to carry out the experiments made it infeasible. Therefore, it was essential to add protease inhibitors. Freezing aqueous gels like mucus can undergo separation in different fractions due to the formation of ice crystals. When thawed, these crystals melt to cause the syneresis of starch gels [68]. However, Macierzanka et al. reported that the freeze–thaw cycle did not alter the permeability and viscosity profiles of the mucus [67]. A lot of other factors influence the physiochemical properties of the mucus. It has been reported that extracellular DNA shed from the enterocytes can significantly contribute to the viscosity and barrier properties of the intestinal mucus layer [32]. Another study found high levels of phosphatidylcholine and phosphatidylethanolamine in young rabbits (< 14 days age), while in adult rabbits (> 28 days age), lysophosphatidylcholine and lysophosphatidylethanolamine were higher [69]. In rats, mucus in early developmental age (5-day-old) had lower amounts of protein, mucin, and DNA when compared to 21-day-old [31]. All these factors affect the physiochemical properties of intestinal mucus. Mucus also contains a variety of immune mediators like mucins, immunoglobulins, antimicrobial peptide, defensins, lysozymes, and reactive oxygen species [20], and their dynamics in young and weaned pigs can also provide an in-depth idea of the virus-blocking effect of mucus.

To check these compositional changes in mucus between the two age-groups, a proteomics analysis was performed, which showed an inherent relatedness of the three replicates per age group through Spearman correlation. Both age-groups shared the majority of 5679 identified proteins. In 3-day mucus, significantly high expressions of IgG-like domains and alpha-casein were found. Both these proteins are indicative of passive immunity transferred through colostrum [70, 71]. In 3-week mucus, protein kinases and ribosomal proteins were expressed significantly more than 3-day mucus. These proteins are important during the growing phase of animals due to their involvement in protein translation and phosphorylation [72, 73]. MUC2, the main mucin of intestinal mucus, was higher in 3-week mucus and could play a role in blocking the viral infection in older pigs, as mucins have been shown to inhibit coronavirus infection in a glycan-dependent manner [74]. Interestingly, MUC13 was significantly more expressed in 3-day mucus. This transmembrane mucin is highly expressed on the apical surface of enterocytes, and its role in negatively regulating the tight junction proteins and intestinal epithelial barrier integrity through protein kinase C has been recently identified [75]. This can also explain the increased viral susceptibility of 3-day-old pigs as epithelial integrity is decreased because of MUC13 activity. APN, the main receptor for TGEV/PRCV [12], had a higher expression in 3-day mucus. This is somewhat confusing as in our previous study using fluorescence microscopy, we found a lower number of APN expressing cells in the intestines of 3-day-old pigs compared to 3-week-old [27]. These seemingly contrasting results may be explained by a higher expression per cell and release of soluble APN in the intestinal lumen of 3-day-old pigs. This soluble APN in mucus may drive TGEV towards susceptible epithelial cells. Cytoskeletal keratin was more expressed in 3-week mucus, and although there are no reports of the interaction of TGEV/PRCV with host cytoskeleton, certain coronaviruses target these proteins for cell entry [76]. Hence, a detailed cytoskeletal profile of the coronavirus target cells can provide useful information. A higher expression of trypsin in 3-day mucus was observed, which is known to increase the infectivity of coronaviruses like SARS-CoV2 by cleaving the S protein [77]. This may also explain the higher infectivity in 3-day-old pigs as compared to 3-week-old. In terms of mucins, the sugar chains are mainly made up of *O*-glycans [78]. In the proteomics analysis, expression of protein *O*-glucosyltransferase 1 was higher in 3-week mucus. This enzyme is important during protein glycosylation as it adds a glucose molecule to the serine residues of proteins, including mucins, providing stability to the epidermal growth factor-like repeats and aiding in trafficking [79]. As glycans are known to inhibit coronavirus infections [74], a higher expression of these sugar transferases could explain the changes in sugar moieties of mucins between different age groups. Hence, the findings of proteomics analysis must be combined with glycomic and transcriptomic analyses for clarifying the broader picture in terms of changes occurring in intestinal mucus during the first few weeks of the animal’s life.

Physical properties of the mucus, like rheology and pore size, are also crucial in this aspect, as they can provide insights into the structural changes in mucus before and after weaning. A previous study from our lab used Atomic Force Microscopy (AFM) to measure the pore size of porcine respiratory mucus [38]. This technique can generate a three-dimensional image of the mucus surface because of the cantilever deflection due to the attractive and repulsive forces from interactions between the cantilever tip and the mucus surface. Mucus rheology is also highly variable, and as discussed before, its gel-solution dynamics change with changes in pH [65]. Collectively, it gives much scope to study these physiochemical parameters of intestinal mucus from different ages of animals concerning intestinal pathogens.

It can be concluded from the present study that porcine ex-vivo intestinal mucus has an age-dependent blocking effect of TGEV. This effect was checked indirectly using a “short duration diffusion” analysis by SPT, “long duration diffusion” analysis, and directly by the TGEV infection of ST cells using a modified IPMA. However, to analyze the active blocking mediators, like the immunomodulatory factors in the mucus, further studies are needed at the molecular level to check age-dependent compositional changes in the mucus. TGEV was used in this study due to ease in laboratory cell culturing and lipophilic labeling. These techniques can now be adapted for various viruses of medical and veterinary importance. It was essential to perform the experiments with mucus from pigs originating from a PRCV/TGEV negative farm, as anti-PRCV/TGEV antibodies were blocking infection, complicating the conclusions that could be made (unpublished data).

## Supplementary Information

**Additional file 1. Details of statistical significance.** Figure 3; Annex A. Detailed comparisons between particles/virions concerning mucus and water in short-duration diffusion by SPT. Figure 3; Annex B. Detailed comparisons between mucus/water concerning particles in long-duration diffusion by SPT. Figure 4; Annex A. Detailed comparisons between particles/virions concerning time points and temperature in long-duration diffusion assay. Figure 4; Annex B. Detailed comparisons between particles concerning the age groups in long-duration diffusion assay. Figure 5; Annex A. Detailed IPMA comparisons within the inoculum/overlay setups of 3-day mucus, 3-week mucus, CMC, and DMEM after 24 h of infection. Figure 5; Annex B. Detailed IPMA comparisons between the 3-day mucus, 3-week mucus, CMC, and DMEM overlay/inoculum setups after 24 h of infection.

**Additional file 2. Detailed proteomics parameters of identified proteins.** Sheet 1 contains the iBAQ values of all identified proteins of the three animals per age group. Sheet 2 provides the statistical significance between age-groups for the identified proteins.

## Data Availability

All relevant data has been included in the main text. Unpublished data are available from the corresponding author on reasonable request.
