# Supplementary material for: Porcine ex-vivo intestinal mucus has age-dependent blocking activity against transmissible gastroenteritis virus
Source: Vet Res. 2024 Sep 20;55:113. doi: 10.1186/s13567-024-01374-y (PMC11414132; doi:10.1186/s13567-024-01374-y)
Supplement: Supplementary file 1 — Additional file 1. Details of statistical significance. Figure 3; Annex A. Detailed comparisons between particles/virions concerning mucus and water in short-duration diffusion by SPT. Figure 3; Annex B. Detailed comparisons between mucus/water concerning particles in long-duration diffusion by SPT. Figure 4; Annex A. Detailed comparisons between particles/virions concerning time points and temperature in long-duration diffusion assay. Figure 4; Annex B. Detailed comparisons between particles concerning the age groups in long-duration diffusion assay. Figure 5; Annex A. Detailed IPMA comparisons within the inoculum/overlay setups of 3-day mucus, 3-week mucus, CMC, and DMEM after 24 h of infection. Figure 5; Annex B. Detailed IPMA comparisons between the 3-day mucus, 3-week mucus, CMC, and DMEM overlay/inoculum setups after 24 h of infection. [file 13567_2024_1374_MOESM1_ESM.docx]

**Additional file 1. Details of statistical significance.**

Figure 3 - Annex A: Detailed comparisons between particles/virions concerning mucus and water in short-duration diffusion by SPT.

| **Age group** | **Temperature** | **Comparison** | **Significance** | ***p*-value** |
| --- | --- | --- | --- | --- |
| 3-days | 20 °C | Carboxylated (-) vs PEGylated (=) | ns | 0.2982 |
|  |  | Carboxylated (-) vs Amide-modified (+) | ** | 0.0013 |
|  |  | Carboxylated (-) vs TGEV (-) | **** | <0.0001 |
|  |  | PEGylated (=) vs Amide-modified (+) | **** | <0.0001 |
|  |  | PEGylated (=) vs TGEV (-) | **** | <0.0001 |
|  |  | Amide-modified (+) vs TGEV (-) | ns | 0.2389 |
|  | 37 °C | Carboxylated (-) vs PEGylated (=) | *** | 0.0010 |
|  |  | Carboxylated (-) vs Amide-modified (+) | **** | <0.0001 |
|  |  | Carboxylated (-) vs TGEV (-) | *** | 0.0008 |
|  |  | PEGylated (=) vs Amide-modified (+) | **** | <0.0001 |
|  |  | PEGylated (=) vs TGEV (-) | **** | <0.0001 |
|  |  | Amide-modified (+) vs TGEV (-) | * | 0.0168 |
| 3-weeks | 20 °C | Carboxylated (-) vs PEGylated (=) | ** | 0.0086 |
|  |  | Carboxylated (-) vs Amide-modified (+) | ** | 0.0041 |
|  |  | Carboxylated (-) vs TGEV (-) | **** | <0.0001 |
|  |  | PEGylated (=) vs Amide-modified (+) | **** | <0.0001 |
|  |  | PEGylated (=) vs TGEV (-) | **** | <0.0001 |
|  |  | Amide-modified (+) vs TGEV (-) | *** | 0.0002 |
|  | 37 °C | Carboxylated (-) vs PEGylated (=) | **** | <0.0001 |
|  |  | Carboxylated (-) vs Amide-modified (+) | ns | 0.0685 |
|  |  | Carboxylated (-) vs TGEV (-) | * | 0.0388 |
|  |  | PEGylated (=) vs Amide-modified (+) | **** | <0.0001 |
|  |  | PEGylated (=) vs TGEV (-) | **** | <0.0001 |
|  |  | Amide-modified (+) vs TGEV (-) | ns | 0.9932 |
| Water | 20 °C | Carboxylated (-) vs PEGylated (=) | ns | 0.2924 |
|  |  | Carboxylated (-) vs Amide-modified (+) | **** | <0.0001 |
|  |  | Carboxylated (-) vs TGEV (-) | **** | <0.0001 |
|  |  | PEGylated (=) vs Amide-modified (+) | ** | 0.0018 |
|  |  | PEGylated (=) vs TGEV (-) | *** | 0.0004 |
|  |  | Amide-modified (+) vs TGEV (-) | ns | 0.9421 |
|  | 37 °C | Carboxylated (-) vs PEGylated (=) | ns | 0.4369 |
|  |  | Carboxylated (-) vs Amide-modified (+) | **** | <0.0001 |
|  |  | Carboxylated (-) vs TGEV (-) | **** | <0.0001 |
|  |  | PEGylated (=) vs Amide-modified (+) | **** | <0.0001 |
|  |  | PEGylated (=) vs TGEV (-) | **** | <0.0001 |
|  |  | Amide-modified (+) vs TGEV (-) | ns | 0.0755 |

**p* < 0.05, ** *p* < 0.01, *** *p* < 0.001, *** *p* < 0.001 or ns = non-significant

Figure 3 - Annex B: Detailed comparisons between mucus/water concerning particles in long-duration diffusion by SPT.

| **Temperature** | **Particle/virion** | **Comparison** | **Significance** | ***p*-value** |
| --- | --- | --- | --- | --- |
| 20 °C | Carboxylated  (-) | 3-day mucus vs 3-week mucus | ns | 0.0820 |
|  |  | 3-day mucus vs Water | **** | <0.0001 |
|  |  | 3-week mucus vs Water | **** | <0.0001 |
|  | PEGylated  (=) | 3-day mucus vs 3-week mucus | ns | 0.8621 |
|  |  | 3-day mucus vs Water | ns | 0.2719 |
|  |  | 3-week mucus vs Water | ns | 0.1102 |
|  | Amide-modified  (+) | 3-day mucus vs 3-week mucus | ns | 0.1898 |
|  |  | 3-day mucus vs Water | ** | 0.0049 |
|  |  | 3-week mucus vs Water | **** | <0.0001 |
|  | TGEV  (-) | 3-day mucus vs 3-week mucus | *** | 0.0002 |
|  |  | 3-day mucus vs Water | *** | 0.0002 |
|  |  | 3-week mucus vs Water | **** | <0.0001 |
| 37 °C | Carboxylated  (-) | 3-day mucus vs 3-week mucus | *** | 0.0003 |
|  |  | 3-day mucus vs Water | *** | 0.0006 |
|  |  | 3-week mucus vs Water | **** | <0.0001 |
|  | PEGylated  (=) | 3-day mucus vs 3-week mucus | * | 0.0276 |
|  |  | 3-day mucus vs Water | ns | 0.2652 |
|  |  | 3-week mucus vs Water | ns | 0.4806 |
|  | Amide-modified  (+) | 3-day mucus vs 3-week mucus | ns | 0.8845 |
|  |  | 3-day mucus vs Water | *** | 0.0002 |
|  |  | 3-week mucus vs Water | *** | 0.0007 |
|  | TGEV  (-) | 3-day mucus vs 3-week mucus | * | 0.0150 |
|  |  | 3-day mucus vs Water | ns | 0.5746 |
|  |  | 3-week mucus vs Water | ns | 0.1275 |

* *p* < 0.05, ** *p* < 0.01, *** *p* < 0.001, *** *p* < 0.001 or ns = non-significant

Figure 4 - Annex A: Detailed comparisons between particles/virions concerning time points and temperature in long-duration diffusion assay.

| **Age-group** | **Time points and temperature** | **Comparison** | **Significance** | ***p*-value** |
| --- | --- | --- | --- | --- |
| 3-days | 10 minutes  @  4 °C | Carboxylated (-) vs PEGylated (=) | **** | <0.0001 |
|  |  | Carboxylated (-) vs Amide-modified (+) | **** | <0.0001 |
|  |  | Carboxylated (-) vs TGEV (-) | ns | 0.6088 |
|  |  | PEGylated (=) vs Amide-modified (+) | ** | 0.0037 |
|  |  | PEGylated (=) vs TGEV (-) | *** | 0.0002 |
|  |  | Amide-modified (+) vs TGEV (-) | **** | <0.0001 |
|  | 30 minutes  @  4 °C | Carboxylated (-) vs PEGylated (=) | **** | <0.0001 |
|  |  | Carboxylated (-) vs Amide-modified (+) | **** | <0.0001 |
|  |  | Carboxylated (-) vs TGEV (-) | **** | <0.0001 |
|  |  | PEGylated (=) vs Amide-modified (+) | ns | 0.0859 |
|  |  | PEGylated (=) vs TGEV (-) | **** | <0.0001 |
|  |  | Amide-modified (+) vs TGEV (-) | ** | 0.0015 |
|  | 10 minutes  @  37 °C | Carboxylated (-) vs PEGylated (=) | ns | 0.9376 |
|  |  | Carboxylated (-) vs Amide-modified (+) | ** | 0.0012 |
|  |  | Carboxylated (-) vs TGEV (-) | ns | 0.0778 |
|  |  | PEGylated (=) vs Amide-modified (+) | ** | 0.0039 |
|  |  | PEGylated (=) vs TGEV (-) | * | 0.0256 |
|  |  | Amide-modified (+) vs TGEV (-) | **** | <0.0001 |
|  | 30 minutes  @  37 °C | Carboxylated (-) vs PEGylated (=) | ns | 0.4037 |
|  |  | Carboxylated (-) vs Amide-modified (+) | ns | 0.4531 |
|  |  | Carboxylated (-) vs TGEV (-) | ns | 0.1627 |
|  |  | PEGylated (=) vs Amide-modified (+) | ns | 0.9997 |
|  |  | PEGylated (=) vs TGEV (-) | ns | 0.9289 |
|  |  | Amide-modified (+) vs TGEV (-) | ns | 0.8967 |
| 3-weeks | 10 minutes  @  4 °C | Carboxylated (-) vs PEGylated (=) | **** | <0.0001 |
|  |  | Carboxylated (-) vs Amide-modified (+) | **** | <0.0001 |
|  |  | Carboxylated (-) vs TGEV (-) | ns | 0.2800 |
|  |  | PEGylated (=) vs Amide-modified (+) | **** | <0.0001 |
|  |  | PEGylated (=) vs TGEV (-) | **** | <0.0001 |
|  |  | Amide-modified (+) vs TGEV (-) | **** | <0.0001 |
|  | 30 minutes  @  4 °C | Carboxylated (-) vs PEGylated (=) | ns | 0.0657 |
|  |  | Carboxylated (-) vs Amide-modified (+) | **** | <0.0001 |
|  |  | Carboxylated (-) vs TGEV (-) | **** | <0.0001 |
|  |  | PEGylated (=) vs Amide-modified (+) | **** | <0.0001 |
|  |  | PEGylated (=) vs TGEV (-) | *** | 0.0004 |
|  |  | Amide-modified (+) vs TGEV (-) | ns | 0.3453 |
|  | 10 minutes  @  37 °C | Carboxylated (-) vs PEGylated (=) | ns | 0.0797 |
|  |  | Carboxylated (-) vs Amide-modified (+) | ** | 0.0035 |
|  |  | Carboxylated (-) vs TGEV (-) | ns | 0.9179 |
|  |  | PEGylated (=) vs Amide-modified (+) | **** | <0.0001 |
|  |  | PEGylated (=) vs TGEV (-) | * | 0.0233 |
|  |  | Amide-modified (+) vs TGEV (-) | * | 0.0128 |
|  | 30 minutes  @  37 °C | Carboxylated (-) vs PEGylated (=) | * | 0.0236 |
|  |  | Carboxylated (-) vs Amide-modified (+) | *** | 0.0005 |
|  |  | Carboxylated (-) vs TGEV (-) | ns | 0.2197 |
|  |  | PEGylated (=) vs Amide-modified (+) | ns | 0.2769 |
|  |  | PEGylated (=) vs TGEV (-) | ns | 0.6238 |
|  |  | Amide-modified (+) vs TGEV (-) | * | 0.0317 |

* *p* < 0.05, ** *p* < 0.01, *** *p* < 0.001, *** *p* < 0.001 or ns = non-significant

Figure 4 - Annex B: Detailed comparisons between particles concerning the age groups in long-duration diffusion assay.

| **Time point** | **Group** | **Comparison** | **Significance** | ***p*-value** |
| --- | --- | --- | --- | --- |
| 10 minutes | 3-day mucus vs  3-week mucus  @  4 °C | Between carboxylated (-) particles | ** | 0.0062 |
|  |  | Between PEGylated (=) particles | ** | 0.0025 |
|  |  | Between amide-modified (+) particles | ns | 0.9173 |
|  |  | Between TGEV (-) | **** | <0.0001 |
|  | 3-day mucus vs  3-week mucus  @  37 °C | Between carboxylated (-) particles | **** | <0.0001 |
|  |  | Between PEGylated (=) particles | **** | <0.0001 |
|  |  | Between amide-modified (+) particles | **** | <0.0001 |
|  |  | Between TGEV (-) | ** | 0.0083 |
| 30 minutes | 3-day mucus vs  3-week mucus  @  4 °C | Between carboxylated (-) particles | **** | <0.0001 |
|  |  | Between PEGylated (=) particles | **** | <0.0001 |
|  |  | Between amide-modified (+) particles | **** | <0.0001 |
|  |  | Between TGEV (-) | **** | <0.0001 |
|  | 3-day mucus vs  3-week mucus  @  37 °C | Between carboxylated (-) particles | **** | <0.0001 |
|  |  | Between PEGylated (=) particles | **** | <0.0001 |
|  |  | Between amide-modified (+) particles | **** | <0.0001 |
|  |  | Between TGEV (-) | **** | <0.0001 |

* *p* < 0.05, ** *p* < 0.01, *** *p* < 0.001, *** *p* < 0.001 or ns = non-significant

Figure 5 - Annex A: Detailed IPMA comparisons within the inoculum/overlay setups of 3-day mucus, 3-week mucus, CMC, and DMEM after 24 hours of infection

| **Setup** | **Comparison** | **Significance** | ***p*-value** |
| --- | --- | --- | --- |
| Overlay | 50% 3-day mucus vs 50% 3-week mucus | ns | 0.4195 |
|  | 50% 3-day mucus vs 5% 3-day mucus | * | 0.0106 |
|  | 50% 3-day mucus vs 5% 3-week mucus | ns | 0.9934 |
|  | 50% 3-day mucus vs 0.94% CMC | ns | 0.0817 |
|  | 50% 3-day mucus vs DMEM | **** | <0.0001 |
|  | 50% 3-week mucus vs 5% 3-day mucus | **** | <0.0001 |
|  | 50% 3-week mucus vs 5% 3-week mucus | ns | 0.1762 |
|  | 50% 3-week mucus vs 0.94% CMC | ns | 0.9300 |
|  | 50% 3-week mucus vs DMEM | **** | <0.0001 |
|  | 5% 3-day mucus vs 5% 3-week mucus | * | 0.0371 |
|  | 5% 3-day mucus vs 0.94% CMC | **** | <0.0001 |
|  | 5% 3-day mucus vs DMEM | ** | 0.0040 |
|  | 5% 3-week mucus vs 0.94% CMC | * | 0.0249 |
|  | 5% 3-week mucus vs DMEM | **** | <0.0001 |
|  | 0.94% CMC vs DMEM | **** | <0.0001 |
| Inoculum | 50% 3-day mucus vs 50% 3-week mucus | * | 0.0153 |
|  | 50% 3-day mucus vs 5% 3-day mucus | ns | 0.1398 |
|  | 50% 3-day mucus vs 5% 3-week mucus | ns | 0.1898 |
|  | 50% 3-day mucus vs 0.94% CMC | *** | 0.0008 |
|  | 50% 3-day mucus vs DMEM | **** | <0.0001 |
|  | 50% 3-week mucus vs 5% 3-day mucus | **** | <0.0001 |
|  | 50% 3-week mucus vs 5% 3-week mucus | ns | 0.8324 |
|  | 50% 3-week mucus vs 0.94% CMC | ns | 0.8229 |
|  | 50% 3-week mucus vs DMEM | **** | <0.0001 |
|  | 5% 3-day mucus vs 5% 3-week mucus | *** | 0.0005 |
|  | 5% 3-day mucus vs 0.94% CMC | **** | <0.0001 |
|  | 5% 3-day mucus vs DMEM | *** | 0.0003 |
|  | 5% 3-week mucus vs 0.94% CMC | ns | 0.1890 |
|  | 5% 3-week mucus vs DMEM | **** | <0.0001 |
|  | 0.94% CMC vs DMEM | **** | <0.0001 |

* *p* < 0.05, ** *p* < 0.01, *** *p* < 0.001, *** *p* < 0.001 or ns = non-significant

Figure 5 - Annex B: Detailed IPMA comparisons between the 3-day mucus, 3-week mucus, CMC, and DMEM overlay/inoculum setups after 24 hours of infection.

| **Comparison between overlay and inoculum setups in:** | **Significance** | ***p*-value** |
| --- | --- | --- |
| 50% 3-day mucus | ns | 0.6310 |
| 50% 3-week mucus | * | 0.0238 |
| 5% 3-day mucus | ns | 0.0783 |
| 5% 3-week mucus | ** | 0.0010 |
| 0.94% CMC | * | 0.0123 |
| DMEM | ns | 0.5818 |

* *p* < 0.05, ** *p* < 0.01, or ns = non-significant
